# Supplementary material for: Impaired Function of Solute Carrier Family 19 Leads to Low Folate Levels and Lipid Droplet Accumulation in Hepatocytes
Source: Biomedicines. 2023 Jan 31;11(2):337. doi: 10.3390/biomedicines11020337 (PMC9953281; doi:10.3390/biomedicines11020337)
Supplement: Supplementary file 1 [file biomedicines-11-00337-s001.zip › AinaraCano-etal_Supplementary Figures_S1toS4.pdf]

Figure S1: *SLC19A1* knockdown THLE2 cells after puromycin selection. Upper graph corresponds to the levels of *SLC19A1* transcript detected by qPCR and the lower images to a western to detect levels of SLC19A1/RFC1 protein.

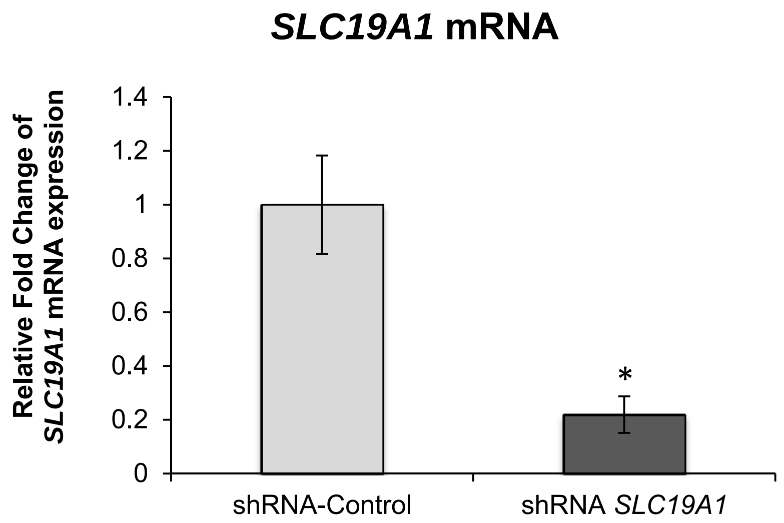

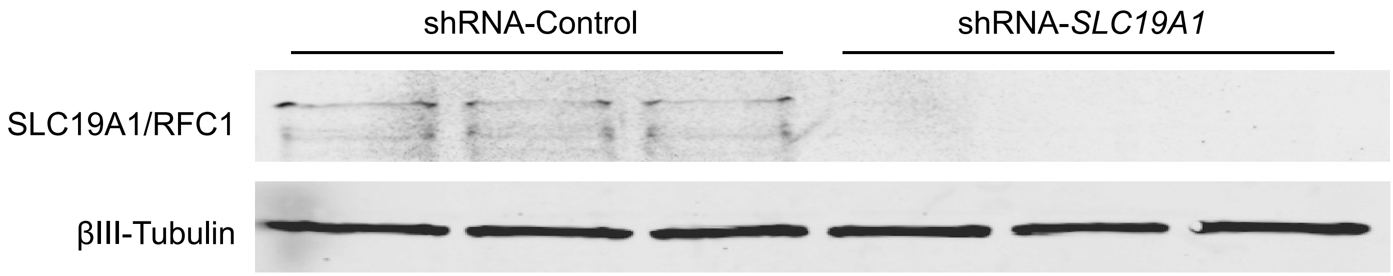

Figure S2: PCA scores plot. In the **PCA scores plot** each icon represents the metabolome hallmark per cell culture made up with 484 metabolites. Silenced cells (*SLC19A1*-KD) are located on the right side of the plot while the controls are located on the left. The first principal component explain 80% of the variation of the data. A=1, R^2X= 0.791, Q^2=0.695

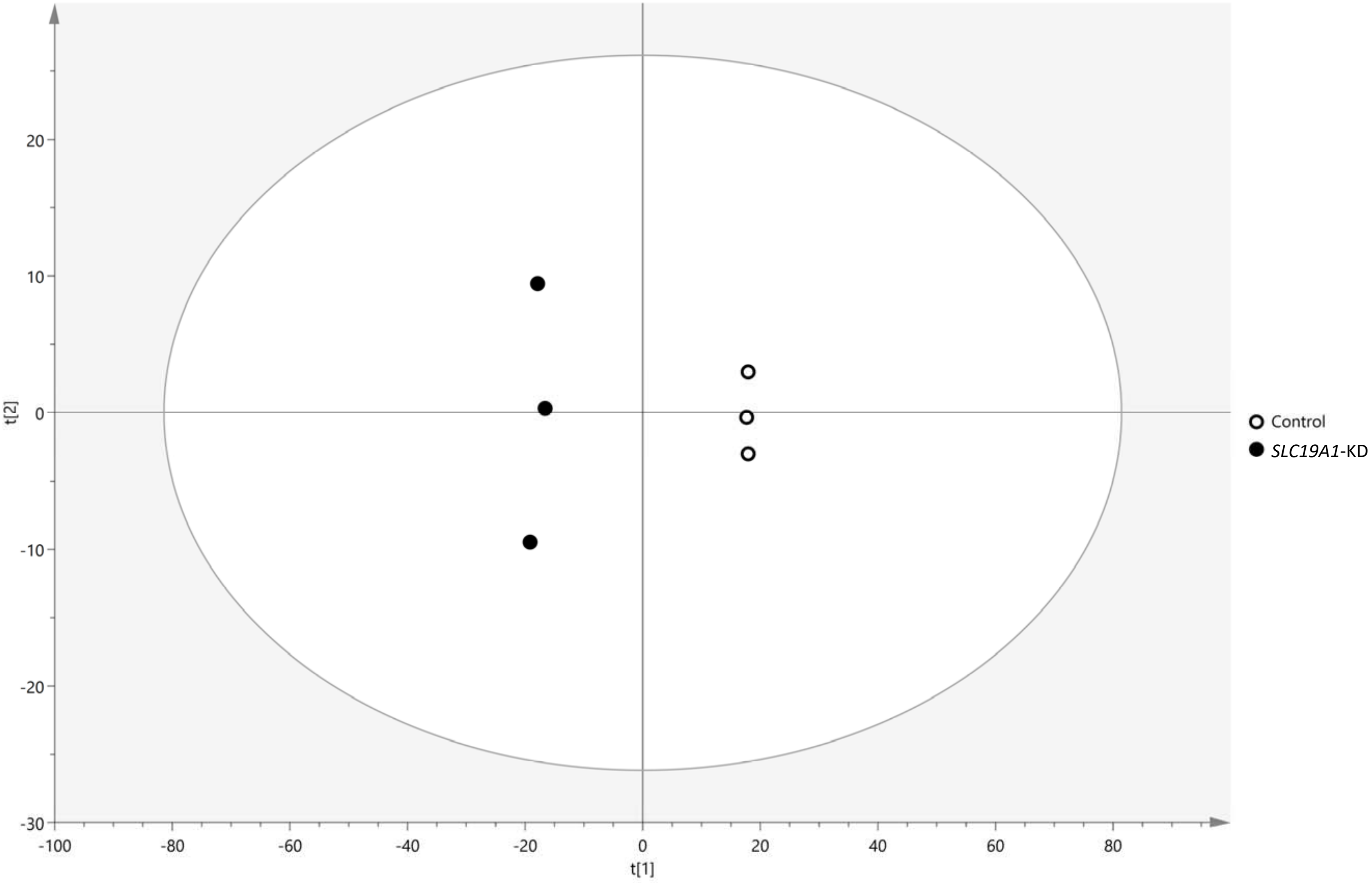

Figure S3: PCA Loadings plot. The majority of the species that increase in *SLC19A1*-KD hepatocytes belong to the chemical groups of triglycerides (TG), primary fatty amides (FAA), and oxidized fatty acids (OxFA). In contrast, a large amount of lysophosphatidylethanolamines (LPE) and amino acids (AA) decrease in RFC1 silenced cells when compared to controls.

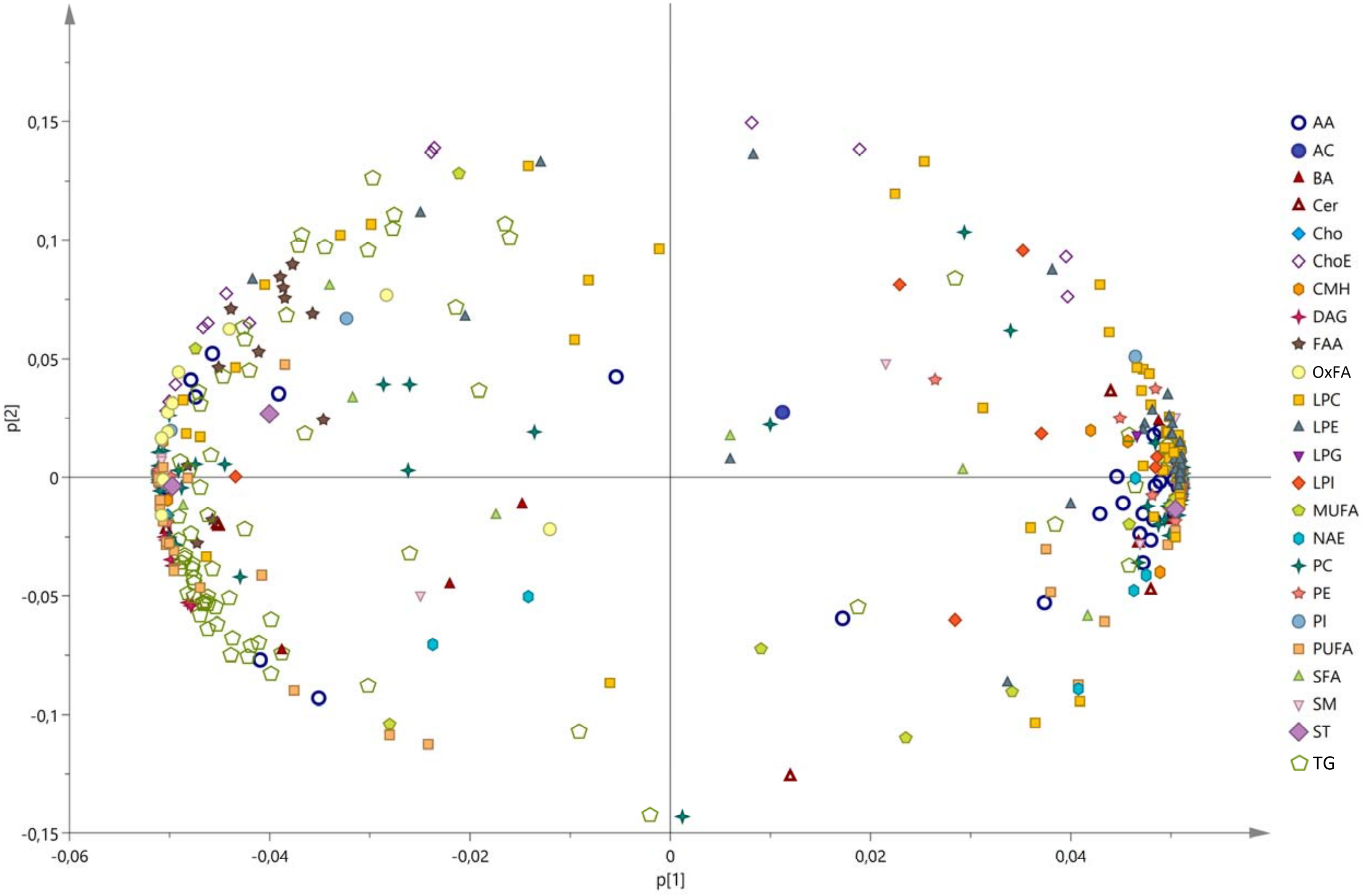

Figure S4-A

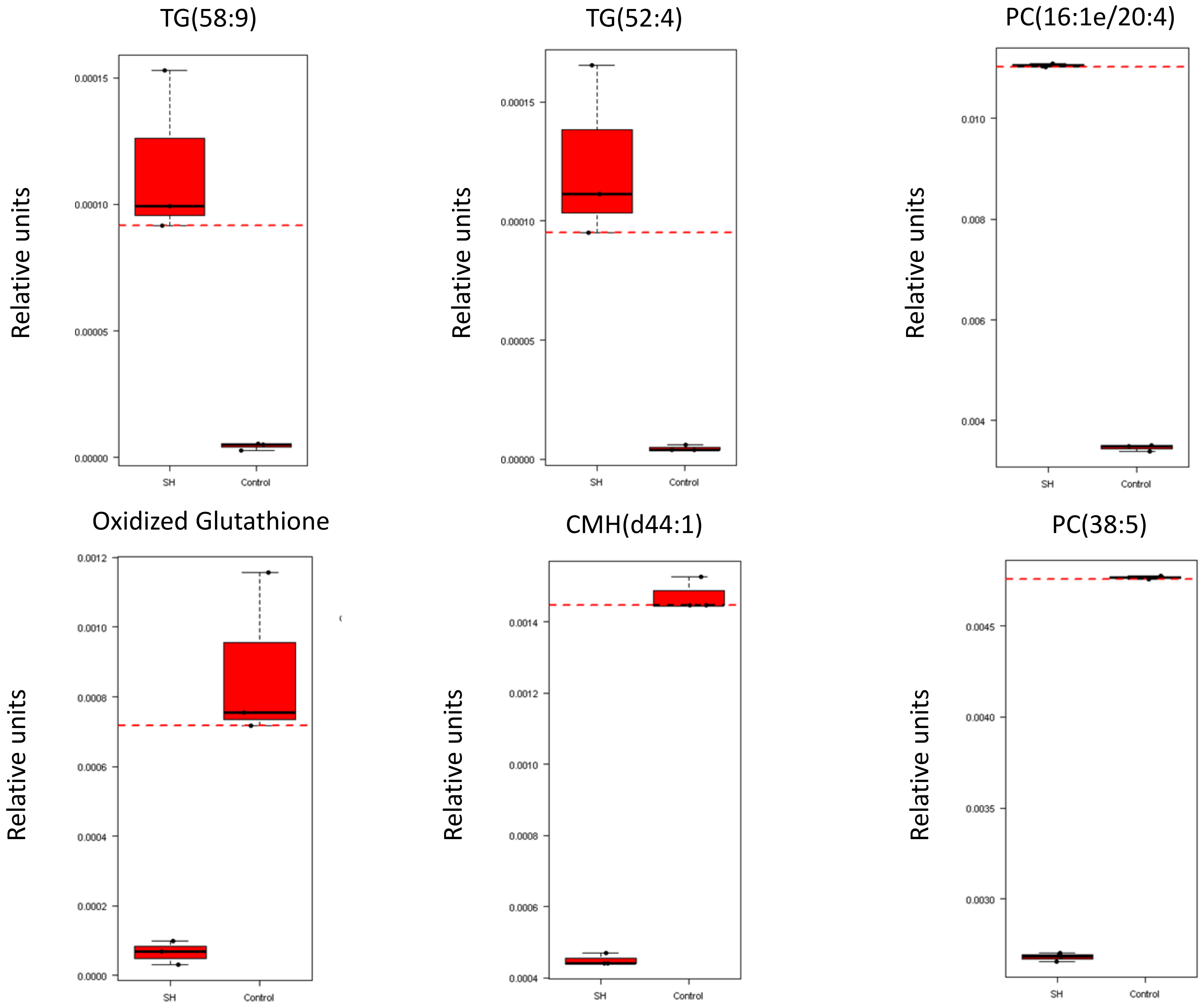
TG(58:9)
Relative units
0.00015
0.00010
0.00005
0.00000
SH
Control
TG(52:4)
Relative units
0.00015
0.00010
0.00005
0.00000
SH
Control
PC(16:1e/20:4)
Relative units
0.010
0.008
0.006
0.004
SH
Control
Oxidized Glutathione
Relative units
0.0012
0.0010
0.0008
0.0006
0.0004
0.0002
0.0000
SH
Control
CMH(d44:1)
Relative units
0.0014
0.0012
0.0010
0.0008
0.0006
0.0004
SH
Control
PC(38:5)
Relative units
0.0045
0.0040
0.0035
0.0030
SH
Control

Figure S4-B

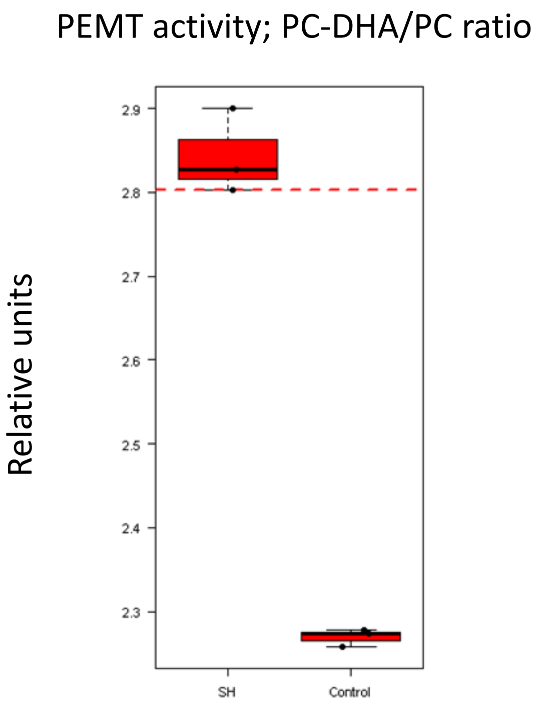

Supplementary Figure S4. Boxplots of the relative concentrations of the significantly altered metabolites (S4-A) and the ratio of phosphatidylcholines (PC) containing docosahexanoic acid to total PC (S4-B), which represents the potential activity of phosphatidylethanolamine N-methyltransferase (PEMT) enzyme in THLE hepatocytes according to the Student´s t test. Data are shown in the Supplementary Excel file “Supplementary Excel file”. Y axes represent relative units since data were semiquantified. The bar plots show the normalized values. The boxes range from the 25% and the 75% percentiles; the 5% and 95% percentiles are indicated as error bars; single data points are indicated by dots. Medians are indicated by horizontal lines within each box.
