# Supplementary material for: Impaired Function of Solute Carrier Family 19 Leads to Low Folate Levels and Lipid Droplet Accumulation in Hepatocytes
Source: Biomedicines. 2023 Jan 31;11(2):337. doi: 10.3390/biomedicines11020337 (PMC9953281; doi:10.3390/biomedicines11020337)
Supplement: Supplementary file 1 [file biomedicines-11-00337-s001.zip › AinaraCano-etal_Supplementary material_230123.pdf]

Supplementary Material

Impaired Function of Solute Carrier Family 19 Leads To Low Folate Levels And Lipid Droplet Accumulation In Hepatocytes

Ainara Cano [1,2,†], Mercedes Vazquez-Chantada [2,3,†,‡], Javier Conde-Vancells [3], Aintzane Gonzalez-Lahera [4,5], David Mosen-Ansorena [4], Francisco J. Blanco [4,6,§], Karine Clément [7,8,9], Judith Aron-Wisnewsky [7,8,9], Albert Tran [10], Philippe Gual [10], Carmelo García-Monzón [5,11], Joan Caballería [5,12], Azucena Castro [2,ǁ], María Luz Martínez-Chantar [4,5], José M. Mato [4,5], Huiping Zhu [3,¶], Richard H. Finnell [3,**,††] and Ana M. Aransay [4,5,*,**]

Methods:

Genotyping and Association Study

SNP genotyping was performed using predesigned TaqMan Assays (Thermo Fisher Scientific). Twenty nanograms of total genomic DNA were used per 5 µl-reaction that consisted of 1x TaqMan® Genotyping Master Mix (Thermo Fisher Scientific), and 1x of each SNP Genotyping probe (Thermo Fisher Scientific, AHWR941, C_1325139_10 and AHX08A9 for rs1051266, rs3788200, and rs3788190, respectively) in 384 well plates. Amplification and detection were carried out in a 7900HT Fast Real-Time PCR System thermocycler (Thermo Fisher Scientific), set at the following cycling conditions: 10 min at 95ºC and 45 cycles of 15 secs at 92ºC and 1 min at 60ºC.

Analysis of *SLC19A1* expression in liver biopsy samples

Total-RNA extracts were isolated from liver biopsies of control individuals and NAFLD patients by DNA/RNA mini kit (Qiagen).

Retro-transcription (RT) following this protocol:

- 275 ng of total-RNA, 1µl of random primers (Invitrogen) (3µg/µl), 1µl dNTP mix (10mM) and sterile distilled water were added per tube
- Mixture was heated at 65ºC for 5 min and contents were collected by brief centrifugation
- 4µl of 5x First-Strand Buffer, 24µl of 0.1 M DTT and 1µl of RNaseOUT™ (40 units/µl) were added per tube
- Tubes were mixed gently and incubated at 42ºC for 2 min
- 1µl of SuperScriptII™ (200 units, Invitrogen, Cat. No. 10777-019) was added per tube and mixed
- Tubes were incubated at 42ºC for 50 min and 70ºC for 15min in order to inactivate the enzyme
- Then, 17µl of each cDNA product were diluted in 300µl of water.

Primers for measuring the mRNA expression of *SLC19A1* were purchased from Qiagen (QuantiTec® Primer Assay). The mRNA expression of Glyceraldehyde-3-phosphate dehydrogenase (*GAPDH*) and acidic ribosomal phosphoprotein (*ARP*), as housekeeping genes, was analyzed using in-house sets of primers:

Seq (5'-3'):

GAPDH L 12 Hs: GAGTCAACGGATTTGGTCGT
GAPDH R 249 Hs: TTGATTTTGGAGGGATCTCG

ARP L Hs: CGACCTGGAAGTCCAACTAC

ARP R Hs: ATCTGCTGCATCTGCTTG

For the qPCR, reactions were set in triplicates. All reactions were done in a total volume of 20µl including the following reagents:

- For QuantiTec® Primer Assay primer sets (per tube):
  1µl of EvaGreen 20x (Biotisem: 31000)
  10µl of Hot Start MasterMix 2x (Metabion, mi-E8011)
  2µl of Forward and Reverse primers 10X
  5µl of cDNA sample
  2µl of distilled water

- For housekeeping genes primer sets (per tube):
  1µl of EvaGreen 20x (Biotisem: 31000)
  10µl of Hot Start MasterMix 2x (Metabion, mi-E8011)
  0.54 µl of Forward primer 100mM
  0.54 µl of Reverse primer 100mM
  5µl of cDNA sample
  2.92µl of distilled water

An automatic pipetting system (Eppendorff epMottion 5070) was used in order to increase the reproducibility of the assays.

The cycling conditions for qPCR of the cDNA from cases and controls, run in an iCycler Thermal Cycler with iCycler iQ Module (Bio-Rad), were:

- For *SLC19A1*: 40 cycles of 15s at 94ºC, 30s at 61ºC and 30s at 72ºC.
- For housekeeping genes (*GAPDH* and *ARP*): 40 cycles of 15s at 94ºC, 30s at 60ºC and 30s at 72ºC.

After checking the specificity of the PCR products by their melting curves, the ratio ($Ct_{housekeeping}/Ct_{target\ gene}$) was calculated as described by Giulietti *et al.* in https://www.sciencedirect.com/science/article/abs/pii/S1046202301912617. Since in this study the Ct values obtained for both housekeeping genes (*GAPDH* and *ARP*) were very similar, a media value of the 2 housekeeping Cts was used to normalize the expression levels of the targeted genes.

Folate concentration measurement

Folate concentrations within the cultured cells were determined by ELISA as previously described by Salojin *et al.*[1]. Briefly, bovine folate binding protein (FBP) (Sigma) (100 ng/well) was immobilized overnight onto Immulon 2HB microtiter 96 well plates (Thermo Fisher Scientific). Excess protein was removed by washing three times with Tris-Buffered-Saline (TBS). Standards of unlabeled folic acid (0.244-250 ng/mL) in TBS-0.1% Tween-20 (TBS-T, pH 7.3) were used to determine concentrations. Standards and cell lysate (10 µL) were diluted to 50 µL in TBS-TA (PBS-T with 1% weight/volume ascorbate, pH ~ 3.0) and placed in boiling water for 10 minutes. Samples were neutralized with 1 µL of 10% NaOH, checked for neutral pH on test strips, and spun for one minute at 14000 g. Supernatants were mixed 1:4 with horseradish peroxidase-labeled folic acid (Cal-Bioreagents) and incubated with immobilized bovine folate binding protein for 1 hour. Plates were washed 5 times with PBS-T, and the interaction between the printed FBP, folates and FA-HRP conjugate was assayed using chemiluminescent reagent Super signal ELISA Femto (Thermo

Fisher Scientific). Photographs of the plates were taken using the Quansys Biosciences Q-View™ and Pixel signal mean intensities were determined using ImageJ 1.46 g (National Institutes of Health). The pixel intensities of the Folate Standard curve were fitted to a sigmoid curve and folate concentration of samples was determined by extrapolated pixel mean intensities in the standard curve using GraphPad Prism 4 Software.

Pathway-focused gene expression analysis. PCR Arrays in knockdown THLE2-cells

Total RNA was isolated from triplicates of sh*SLC19A1*-KD and shControl THLE2-cells, with the RNeasy Micro Kit (Qiagen). After assessing yield and quality of the RNA with the 2100Bioanalyzer (Agilent Technologies), cDNA synthesis and elimination of genomic DNA was carried out with RT^2first-strand kit (Qiagen). Subsequently, gene expression profiling was performed with the RT^2profiler™ PCR-Array System (PAHS-157Z Fatty liver, Qiagen), RT^2SYBR-Green mastermix and amplification was performed using the Life Technologies 7900HT Fast Real-Time-PCR System thermocycler. Data were evaluated using "RT^2profiler™ PCR-Array data analysis software" (Qiagen), which calculates relative expression using the ΔΔCt method[2]. *RPLP0* and *GAPDH* were selected as reference genes (based on the small variation in Ct values across the included samples).

Reviewing the list of the genes that were found to be differentially expressed, considering thresholds at a fold-change of 1.4 and p-value<0.05, and adding to the list the gene that was silenced, *SLC19A1*, a "Reactome" functional interaction network was built using the Cytoscape software[3]. Linker genes were included in the network as necessary in order to connect all genes. The network was subsequently exported to Gephi (https://gephi.org/), where the ForceAtlas2 (https://github.com/bhargavchippada/forceatlas2) clustering algorithm was applied to the graph, placing interconnected genes together and central genes in the middle of the graph.

Metabolite extraction

In metabolic profiling, there is no single platform or method to analyze the entire metabolome of a biological sample, primarily due to the wide concentration range of metabolites coupled to their extensive chemical diversity (https://www.nature.com/articles/nmeth0211-117.pdf and https://link.springer.com/content/pdf/10.1007/s11306-011-0324-1.pdf?pdf=button). Therefore, metabolite extraction was accomplished by fractionating the cell samples into pools of species with similar physicochemical properties, using appropriate combinations of organic solvents. The following methods were used according to the chemical class of targeted analytes.

Cell pellets were resuspended in cold water and briefly mixed. Proteins were precipitated from the lysed cell samples by adding methanol. After short vortex mixing the samples were spiked with chloroform. Both extraction solvents were spiked with metabolites not detected in unspiked cell extracts (internal standards). Samples were incubated at -20℃ for 30 minutes and after vortex two different phases were collected and analyzed in three different platforms:

Platform 1: Fatty acyls, bile acids, steroids and lysoglycerophospholipids profiling. Supernatants were collected after centrifugation at 16000 x g for 15 minutes, dried,

reconstituted in methanol, resuspended for 20 minutes and centrifuged (16000 x g for 5 minutes) before being transferred to vials for UHPLC-MS analysis.
Platform 2: Glycerolipids, cholesteryl esters, sphingolipids and glycerophospholipids profiling. Cell extracts were mixed with water (pH 9) and after brief vortexing samples were incubated for 1 hour at -20℃. After centrifugation at 16000 x g for 15 minutes, the organic phase was collected. Dried extracts were then reconstituted in acetronitrile / isopropanol (50:50), resuspended for 10 minutes, centrifuged (16000 x g for 5 minutes), and transferred to vials for UHPLC-MS analysis.
Platform 3: *Amino acids profiling*. Aliquots from the extracts prepared for Platform 1 were transferred to microtubes and derivatized for amino acid analysis.

Additionally, two different types of quality control (QC) samples were used to assess the data quality[4]. QC samples are reference serum samples, which are evenly distributed over the batches and extracted and analyzed at the same time as the individual samples. *QC Calibration sample:* used to correct the different response factors between and within batches. *Validation sample:* used to assess how well data pre-processing procedure improved the data quality.

For each of the three analytical platforms, randomized duplicate sample injections were performed, with each of the QC calibration and validation extracts uniformly interspersed throughout the entire batch run.

LC-MS analysis
Three different UHPLC-MS methods were used for each platform; chromatographic separation and mass spectrometric detection conditions employed are fully described in Barr *et al.*[5] and more recently in Manni *et al.*[6].

Each extract was spiked with metabolites not detected in unspiked human serum extracts: tryptophan-d5 (indole-d5), PC(13:0/0:0), NEFA(19:0) and dehydrocholic acid in methanol extract; SM(d18:1/6:0), PE(17:0/17:0), PC(19:0/19:0), TAG(13:0/13:0/13:0), TAG(17:0/17:0/17:0), Cer(d18:1/17:0) and ChoE(12:0) in chloroform/ methanol extract.

A test mixture of standard compounds was analyzed before and after the entire set of randomized, duplicated sample injections in order to examine the retention time stability (generally < 6 s variation, injection-to-injection), mass accuracy [platforms 1 and 3 (generally < 3 ppm for *m/z* 400-1000, and < 1.2 mDa for *m/z* 50-400)] and sensitivity of the system. For each injection batch, the overall quality of the analysis procedure was monitored using five repeated extracts of the QC Validation sample.

Data Pre-Processing
All data were processed using the TargetLynx application manager for MassLynx 4.1 software (Waters Corp., Milford, USA). A set of predefined retention time, mass-to-charge ratio pairs, *Rt- m/z*, corresponding to metabolites included in the analysis are fed into the program. Associated extracted ion chromatograms (mass tolerance window = 0.05 Da) are then peak-detected and noise-reduced in both the LC and MS domains such that only true metabolite related features are processed by the software. A list of chromatographic peak areas is then generated for each sample injection.

For identified metabolites, representative MS detection response curves were generated

using an internal standard for each chemical class included in the analysis. By assuming similar detector response levels for all metabolites belonging to a given chemical class, this allowed a linear detection range to be defined for each variable.

Data Normalization and quality control
Peak intensities for each ion feature included in the analysis were normalized to the sum of the peak intensities within each sample, as described in Manni et al[6]. There was no significant correlation between the sum of the peak intensities used for the intra-batch normalization and the groups being compared in this study. Once normalized, the consistency between duplicate sample injection response values was evaluated, the median%CV value being 5%. The study samples were accompanied by six repeat extracts of a pooled sample, which were used to assess the reproducibility of the analysis process. Following normalization, the concordance between duplicate sample injection response values was assessed. Where coefficients of variation > 15% were found, corresponding sample injection data were returned for manual inspection of the automated integration performed by the TargetLynx software, and modifications performed where appropriate.

Any remaining sample injection variable response zero values in the corrected dataset were replaced with missing values before averaging to form the final dataset that was used for study sample statistical analyses.

Other Statistical Analyses
Shapiro-Wilk test: the Shapiro-Wilk test was used for testing the normality of data. It was applied to each metabolite to detect whether a sample comes from normally distributed population.

Supplementary_References

1. Salojin, K.V.; Cabrera, R.M.; Sun, W.; Chang, W.C.; Lin, C.; Duncan, L.; Platt, K.A.; Read, R.; Vogel, P.; Liu, Q.; et al. A mouse model of hereditary folate malabsorption: deletion of the PCFT gene leads to systemic folate deficiency. *Blood* **2011**, *117*, 4895-4904, doi:10.1182/blood-2010-04-279653. (Reference 30 in the manuscript)
2. Schmittgen, T.D.; Livak, K.J. Analyzing real-time PCR data by the comparative C(T) method. *Nature protocols* **2008**, *3*, 1101-1108. (Reference 31 in the manuscript)
3. Shannon, P.; Markiel, A.; Ozier, O.; Baliga, N.S.; Wang, J.T.; Ramage, D.; Amin, N.; Schwikowski, B.; Ideker, T. Cytoscape: a software environment for integrated models of biomolecular interaction networks. *Genome research* **2003**, *13*, 2498-2504, doi:10.1101/gr.1239303. (Reference 32 in the manuscript)
4. Martinez-Arranz, I.; Mayo, R.; Perez-Cormenzana, M.; Minchole, I.; Salazar, L.; Alonso, C.; Mato, J.M. Enhancing metabolomics research through data mining. *J Proteomics* **2015**, *127*, 275-288, doi:10.1016/j.jprot.2015.01.019. (Reference 35 in the manuscript)
5. Barr, J.; Caballeria, J.; Martinez-Arranz, I.; Dominguez-Diez, A.; Alonso, C.; Muntane, J.; Perez-Cormenzana, M.; Garcia-Monzon, C.; Mayo, R.; Martin-Duce, A.; et al. Obesity-dependent metabolic signatures associated with nonalcoholic fatty liver disease progression. *Journal of proteome research*

2012, *11*, 2521-2532, doi:10.1021/pr201223p. (Reference 33 in the manuscript)

6. Manni, M.M.; Valero, J.G.; Perez-Cormenzana, M.; Cano, A.; Alonso, C.; Goni, F.M. Lipidomic profile of GM95 cell death induced by Clostridium perfringens alpha-toxin. *Chem Phys Lipids* **2017**, *203*, 54-70, doi:10.1016/j.chemphyslip.2017.01.002. (Reference 34 in the manuscript)
